# Efficacy and Safety of Indwelling Pleural Catheters in Management of Hepatic Hydrothorax: A Systematic Review of Literature

**DOI:** 10.7759/cureus.3110

**Published:** 2018-08-06

**Authors:** Muhammad A Baig, Muhammad B Majeed, Bashar M Attar, Zubair Khan, Melchor Demetria, Seema R Gandhi

**Affiliations:** 1 Medicine, John H Stroger J. Hospital of Cook County, Chicago, USA; 2 Medicine, John H. Stroger Jr. Hospital of Cook County, Chicago, USA; 3 Gastroenterology and Hepatology, John H. Stroger Jr. Hospital of Cook County, Chicago, USA; 4 Internal Medicine, University of Toledo Medical Center, Toledo, USA

**Keywords:** hepatic hydrothorax, indwelling pleural catheters, pleural catheters, systematic review

## Abstract

Hepatic hydrothorax (HH) is an infrequent but debilitating and therapeutically challenging complication of advanced liver cirrhosis. As evidence suggests against chest tube placement in HH, many clinicians are reluctant to place indwelling pleural catheters (IPCs) for non-malignant effusions like HH. We aim to study the efficacy and safety of IPCs as an alternative treatment option in our systematic review.

A literature search was conducted using the electronic database engines MEDLINE, PubMed, EMBASE, Ovid, Scopus and Cochrane Library (Cochrane Central Register of Controlled trials and Cochrane Database of Systematic Reviews) from inception to April 2018 to identify published articles and reports addressing outcomes in patients treated for HH with IPCs. The risk of bias was rated for each study using the Cochrane criteria.

The search strategy retrieved 370 papers, of which four case series were selected with a total of 111 patients. After the insertion of IPCs for HH, spontaneous pleurodesis was achieved in 16 (31.4%) out of 51 patients at a mean duration of 73-222 days. As far as secondary outcomes were concerned, the frequency of pneumothorax during or after the procedure was 0 (0%) out of 92 patients, pain at insertion site 12 (20%) out of 60 patients, catheter blockage two (2.9%) out of 68 patients, pleural fluid infection five (4.5%) out of 111 patients and catheter-site cellulitis one (3.1%) out of 32 patients. Re-accumulation of pleural fluid after catheter removal was mentioned in one study, wherein 12 (20%) out of 60 patients developed recurrence of pleural effusion.

We conclude IPCs as an acceptable therapeutic option for the management of refractory pleural effusion in patients with HH. Although trans-jugular intrahepatic portosystemic shunt (TIPS) and liver transplantation are the gold standards for the management of pleural effusion in these patients, cost and availability are the major concerns with these treatment modalities. IPCs are a safe and efficacious alternative with a reasonable rate of spontaneous pleurodesis.

## Introduction and background

Hepatic hydrothorax (HH) is defined as a transudative pleural effusion >500 mL in patients with cirrhosis in the absence of cardiac and pulmonary diseases [1]. In patients with cirrhosis, the incidence of HH is 5% to 10%, and HH occurs typically as an isolated right-sided pleural effusion in 70% to 80% of cases. The relief of dyspnea in patients with HH that is refractory to conservative management remains a clinical challenge [2]. As the majority of these patients have concomitant ascites, the therapeutic aim is typically to treat the ascites with salt restriction and diuretics and provide thoracentesis as needed [3].

It can become increasingly challenging when patients develop diuretic-resistant HH, as the only options available are liver transplantation, trans-jugular intrahepatic portosystemic shunt (TIPS) and indwelling pleural catheter (IPC). Several case series have demonstrated the association of chest tube placement in patients with HH with complications such as major electrolyte imbalances, malnutrition, severe protein loss, acute renal injury, pneumothorax and empyema [4-7]. Given the questionable safety of chest tube placement, many clinicians are reluctant to place IPCs for managing non-malignant effusions like HH [8]. Moreover, limited objective data are available on the efficacy of IPCs in this patient population.

To the best of our knowledge, this is the only systematic review of literature to date detailing the safety and efficacy of IPCs in patients with HH. 

## Review

Materials and methods

A literature search was conducted using the electronic database engines MEDLINE, PubMed, EMBASE, Ovid, Scopus and Cochrane Library (Cochrane Central Register of Controlled Trials and Cochrane Database of Systematic Reviews) from inception to April 2018 to identify published articles and reports addressing outcomes in patients treated for HH with IPCs. The combinations of keywords used were “indwelling pleural catheter” or “PleurX catheter” or “tunneled pleural catheter” or “pleural catheter” or “catheter” and “hepatic hydrothorax”. The reference lists of all eligible studies were reviewed to identify additional studies.

**Figure 1:**
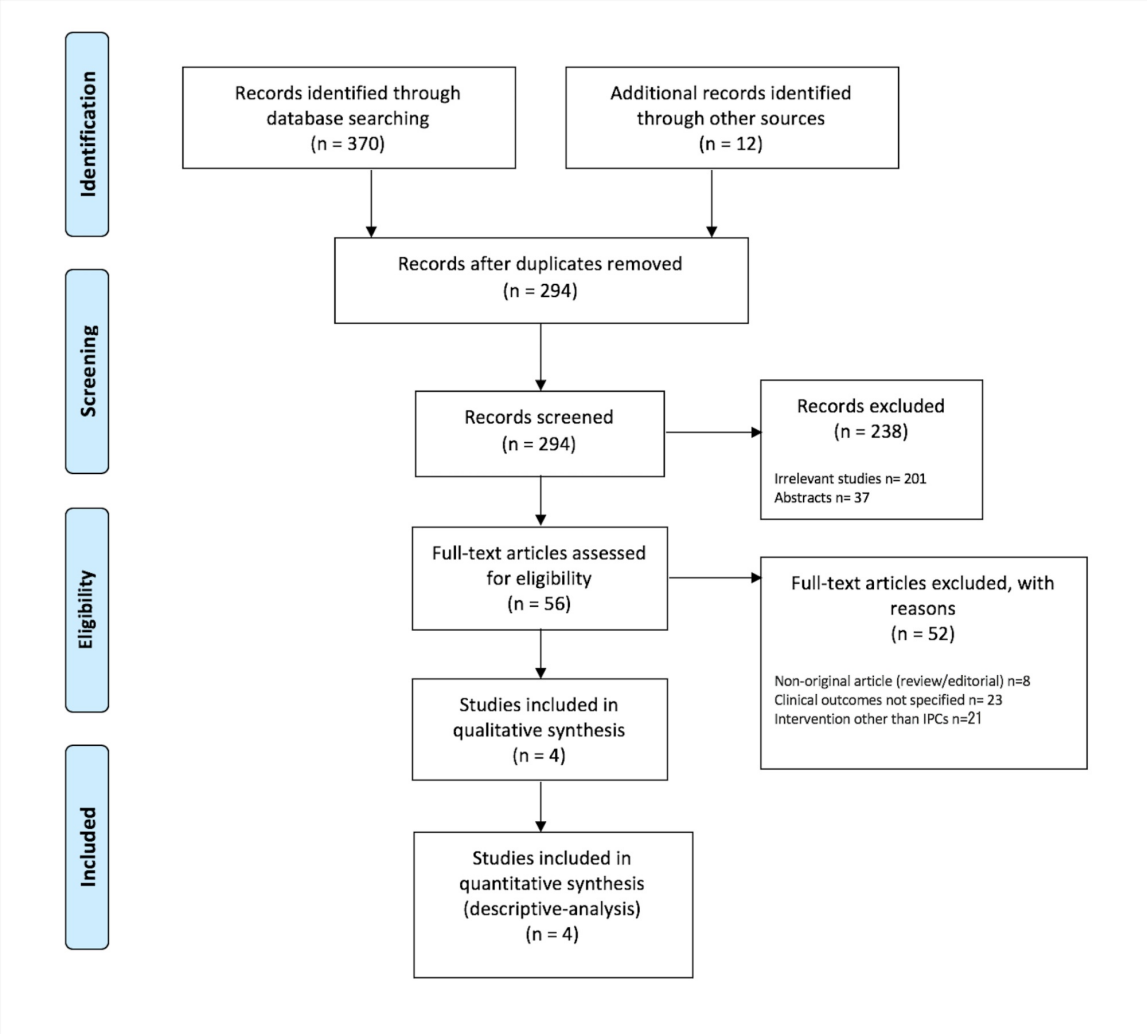
Preferred Reporting Items for Systematic Reviews and Meta-Analyses flow diagram detailing the review process.

Published studies, case series and case reports were eligible for inclusion if they reported the use of IPCs for the management of HH. Articles were excluded if (1) they were not written in English, (2) no outcomes were reported, or (3) they represented single case reports, review articles or studies published as abstracts only. In observational studies using multiple modalities for the management of HH, data from the cohort of patients who underwent IPC placement were collected and analyzed. Two reviewers (MB and MM) independently performed study selection according to the eligibility criteria. Disagreements were resolved by discussion with a third reviewer (ZK). A Preferred Reporting Items for Systematic Reviews and Meta-Analyses flow diagram detailing the review process is shown in Figure 1.

The following data were independently abstracted into a standardized form: study characteristics (study design, primary author, time period of study, year of publication and country of the population studied), characteristics of the study population (total number of patients undergoing IPC placement, age of patients, gender, location of pleural effusion, Child-Pugh Class, Model for End-Stage Liver Disease score and etiology of cirrhosis), type and frequency of complications (pneumothorax, insertion-site pain, blockage of catheter, pleural fluid infection, re-accumulation after catheter removal, catheter-site cellulitis and dislodgement of the catheter) and outcomes of IPCs (spontaneous pleurodesis and mean duration to achieve spontaneous pleurodesis). 

We systematically assessed the quality of the studies using the published criteria [9-10]. To comprehensively assess the quality of clinical studies, we used the Newcastle-Ottawa Scale (NOS) [11]. Again, in the case of a discrepancy in the quality assessment between the two study investigators (MB and MM), the senior investigator (ZK) reviewed the data and an agreement was obtained. The risk of bias was rated for each study by two authors independently, using the Cochrane criteria for randomized controlled trials (RCTs) [12]. Uncontrolled studies were always rated as moderate or high risk of bias (never at low risk of bias) and categorized as follows: moderate risk of bias, when the descriptions of patients, interventions and outcomes of interest were complete and clear throughout the observation period and high risk of bias, when the descriptions of patients, interventions and outcomes were incomplete or unclear, or when follow-up was incomplete.

The primary outcome analyzed in this study was spontaneous pleurodesis which was defined as a decrease in pleural fluid drainage to <50 mL on three consecutive IPC drainage procedures and no evidence of significant effusion on chest ultrasonography, chest radiography, or chest computed tomography (CT) scan. The secondary outcomes included the time to achieve spontaneous pleurodesis, requirement of further pleural drainage after IPC placement and rate of complications (pleural fluid infection, pain at the insertion site, insertion-site cellulitis and pneumothorax).

All the studies included in this systematic review were observational, and hence, a meta-analysis could not be performed. Pooled results on the efficacy and safety outcomes were reported for each outcome as the percentage of patients with the primary or secondary outcome. 

Findings were reported in tables and text, as data could not be pooled in forest plots.

Results

The search strategy described above retrieved a total of 370 published articles. Among these, only four case series were identified to qualify for inclusion, as described in Figure 1. The characteristics of these primary studies are detailed in Table 1. 

**Table 1:** Characteristics of primary studies

Study included	Primary author	Time period of study	Year of publication	Country of population	Study design
	Chalhoub et al. [13]	2003-2009	2011	United States of America	Case series
	Bhatnagar et al. [14]	2007-2013	2013	United Kingdom	Case series
	Sharaf-Eldin et al. [15]	Not reported	2016	Egypt	Case series
	Chen et al. [16]	2010-2015	2016	United States of America	Case series

The characteristics of patients in the included studies are reported in Table 2.

**Table 2:** Characteristics of patients in included studies MELD: model for end-stage liver disease; NASH: nonalcoholic steatohepatitis; PBC: primary biliary cholangitis

VARIABLES	Sharaf-Eldin et al. [15]	Chalhoub et al. [13]	Chen et al. [16]	Bhatnagar et al. [14]	Pooled Analysis
Number of Patients	60	8	24	19	111
Mean Age (years)	42.3	56	59.8	Not Reported	
Male (n)	36	6	Not Reported	Not Reported	42 out of 68 (61.8%)
Female (n)	24	2	Not Reported	Not Reported	26 out of 68 (38.2%)
Right-sided Pleural Effusions (n)	Not Reported	8	21	Not Reported	29 out of 32 (90.6 %)
Left-sided Pleural Effusions (n)	Not Reported	0	3	Not Reported	3 out of 32 (9.4%)
Child-Pugh A (n)	0	Not Reported	Not Reported	Not Reported	
Child-Pugh B (n)	32	Not Reported	Not Reported	Not Reported	
Child-Pugh C (n)	28	Not Reported	Not Reported	Not Reported	
MELD Score	Not Reported	Not Reported	19.4	Not Reported	
Alcoholic Cirrhosis (n)	Not Reported	Not Reported	11	Not Reported	
NASH (n)	Not Reported	Not Reported	6	Not Reported	
Chronic Hepatitis-induced Cirrhosis (n)	Not Reported	Not Reported	6	Not Reported	
PBC Cirrhosis (n)	Not Reported	Not Reported	1	Not Reported	
Cryptogenic Cirrhosis (n)	Not Reported	Not Reported	1	Not Reported	

In total, we investigated data from 111 patients. The mean age of patients was reported in three studies (Chalhoub et al. [13], Chen et al. [16] and Sharaf-Eldin et al. [15]) and ranged from 42 to 59 years. The majority of these patients were male (61.8%). The location of pleural effusion was mentioned in two studies (Chalhoub et al. [13] and Chen et al. [16]) with right-sided predominance (90.6%). Based on the available studies, it was difficult to estimate the duration of IPC in situ as it was not reported in most of the studies. The etiology of the underlying chronic liver disease was reported in only one study (Chen et al. [16]), with alcoholic cirrhosis (45%) and nonalcoholic steatohepatitis (25%) being the most common etiologies. Severity of the liver disease was also described only by Chen et al. [16], which included 32 patients with Child-Pugh class B cirrhosis and 28 patients with Child-Pugh class C cirrhosis. The mean number of thoracentesis before IPC placement was investigated in three studies (Chalhoub et al. [13], Chen et al. [16] and Bhatnagar et al. [14]) and ranged from 3.5 to 4.5 times.

The primary and secondary outcomes are reported in Table 3. 

**Table 3:** Primary and secondary outcomes IPC: indwelling pleural catheter

VARIABLES	Sharaf-Eldin et al. [15]	Chalhoub et al. [13]	Chen et al. [16]	Bhatnagar et al. [14]	Pooled Analysis
Mean number of thoracentesis before IPC (n)	Not reported	3.5	1.9	4.5	
Spontaneous pleurodesis (n)	Not reported	6	8	2	16 out of 51 (31.4%)
Mean time to spontaneous pleurodesis (days)	Not reported	73.6	131.8	222	
Requirement of pleural drainage after IPC	Not reported	0	0	Not reported	0 out of 32 (0%)
Pneumothorax (n)	0	0	0	Not reported	0 out of 92 (0%)
Pain at insertion site (n)	12	Not reported	Not reported	Not reported	12 out of 60 (20%)
Blockage of the catheter (n)	2	0	Not reported	Not reported	2 out of 68 (2.9%)
Pleural fluid infection (n)	0	0	4	1	5 out of 111 (4.5%)
Reaccumulation after catheter removal (n)	12	0	Not reported	Not reported	12 out of 68 (17.6%)
Catheter site cellulitis (n)	Not reported	1	0	Not reported	1 out of 32 (3.1%)
Dislodgement (n)	Not reported	0	Not reported	1	1 out of 27 (3.7%)

Of the 51 patients, 16 (31.4%) achieved spontaneous pleurodesis. The mean duration to achieve spontaneous pleurodesis was described in three studies (Chalhoub et al. [13], Chen et al. [16] and Bhatnagar et al. [14]) and ranged from 73 to 22 days. Further requirement of pleural fluid drainage by other means after IPC placement was described by Chalhoub et al. [13] and Chen et al. [16]. In the 32 patients followed up, none of them required any drainage after IPC placement. As for secondary outcomes, the frequency of pneumothorax during or after the procedure was 0 out of 92 patients (0%); pain at insertion site was reported in 12 out of 60 patients (20%), catheter blockage in two out of 68 patients (2.9%), pleural fluid infection in five out of 111 patients (4.5%) and catheter-site cellulitis in one out of 32 patients (3.1%). Re-accumulation of pleural fluid after catheter removal was mentioned in one study (Sharaf-Eldin et al. [15]), wherein 12 out of 60 patients (20%) developed a recurrence of pleural effusion.

Discussion

The management of patients with cirrhosis and HH can be clinically challenging as limited objective data are available regarding the recommended treatment strategies for HH. These patients are largely managed primarily with salt restriction and diuretics. In a case series by Badillo et al. [17], 83% of patients with HH responded to sodium restriction and diuretics. Among patients with HH, 20% are refractory to diuretics, requiring other modalities for symptomatic control. While awaiting transplant, these options can include repeated therapeutic thoracentesis, pleurodesis, IPCs or TIPS. Patients with HH already have an overall elevated mortality rate compared to those with cirrhosis with the same MELD score [17]. Most patients with HH have Child-Pugh class B or class C cirrhosis and are listed for transplant; however, due to organ scarcity, alternative temporizing therapies are often needed [18-19]. 

An alternative to organ transplantation, TIPS also addresses the underlying pathology causing HH and can be an effective management strategy for recurrent HH [20]. Potential complications of TIPS include thrombosis or stenosis within the shunt. In addition, TIPS can cause hepatic encephalopathy in 15% to 30% of the patients [21]. Also, one-third of the patients continue to require repeated thoracentesis [21-22].

IPCs are widely used in patient populations with refractory pleural effusions secondary to malignancy, congestive heart failure and renal failure [14,23]. IPCs should be considered as a bridging treatment modality between liver transplantation and refractory HH because of its efficacy, safety and low cost.

We analyzed the rate of spontaneous pleurodesis as the primary outcome in our study. In smaller studies on HH, the rate of pleurodesis ranged from 10% to 75% [13-14]. In our pooled analysis, pleurodesis was achieved in 16 out of 51 patients (31.4%). This rate is comparable to the rate of pleurodesis achieved with IPCs in patients with congestive heart failure and malignant pleural effusions. In a systemic review by Patil et al. [24], 162 patients with refractory pleural effusion due to heart failure were managed with IPCs, of which 68 patients (42.1%) achieved spontaneous pleurodesis. The rate of spontaneous pleurodesis in malignant pleural effusions was analyzed by Putnam at al. [25] and Davies et al. [26] and was found to be 46% and 51%, respectively. The mean duration of achieving spontaneous pleurodesis in patients treated for HH with IPC ranged from 73 to 222 days, which is significantly higher than the time taken to achieve pleurodesis in malignant pleural effusions (36 days), as reported in a study by Van Meter et al. [23]. It is hypothesized that the longer time to achieve pleurodesis in HH could be secondary to rapid accumulation of fluid, which prevents the visceral and parietal pleural surfaces to approximate and adhere [27]. The mechanism of pleurodesis with IPC is not well understood, but it is thought to be related to low-grade inflammatory reaction that develops in response to the catheter acting as a foreign body in the pleural space [13].

As secondary outcomes, we analyzed the rate of pleural fluid infection, pneumothorax and catheter-site cellulitis after IPC insertion for HH. In a smaller study on HH by Fortin et al. [28], pleural fluid infection was overestimated to be 16.7%, but as per our review, only five out of 111 patients (4.5%) developed this complication. This rate of pleural fluid infection (4.5%) was much lower than the frequency of pleural fluid infection reported by Davies et al. [26] in patients with malignant pleural effusions (13.4%). However, the rate of pleural fluid infection after IPC placement for HH (4.5%) was found to be slightly higher compared to that in patients with congestive heart failure (1.5%) and renal failure (0%) [14,23]. In our review, there were no reports of pneumothorax among 92 patients with HH. However, in patients with malignant pleural effusion requiring IPCs, the rate of pneumothorax requiring chest tube insertion was reported to be 5.9% [29]. The rate of IPC blockage was also found to be lower in patients with HH (2.9%) as compared to those with malignant pleural effusion (3.7%) [23]. Catheter blockage and insertion-site cellulitis were also found in only one of 32 cases (3.1%) and 12 out of 60 patients (20%), respectively, thus making IPCs a relatively safe therapeutic option for patients with HH. 

Among patients with refractory HH who are not yet able to receive a liver transplant, the use of IPCs may portend better survivals rates as compared to patients managed with TIPS. A review by Xiol et al. [30] presented 56 patients with HH undergoing liver transplantation with a one-year survival post-transplant of 82% and a mean survival of 97 months compared with  40 to 60% survival at 1 year and a mean survival of 7 to 13 months in patients with refractory HH treated with TIPS [31]. Moreover, in a case series by Campos et al. [20], TIPS was associated with 25% mortality at 30 days, and portosystemic encephalopathy was recorded in 66.6% of the cases. In addition to a higher reported mortality, TIPS has a high financial burden for the patient, as compared to an IPC. In a recent study by Penz et al. [32], the overall cost for managing patients with IPCs was estimated to be $4,993 which is significantly lower than the expenditure for TIPS in which the mean hospital cost was $44,901 ± $54,565 [33]. IPCs are a safe, economical and efficacious alternative to TIPS for patients who are awaiting liver transplantation and have recurrent HH. 

Study limitations

Our systematic review has several limitations. First, despite performing a comprehensive literature search in multiple well-established databases, independently conducted by two reviewers, and careful cross-referencing, we cannot exclude the possibility of having missed a relevant study. Second, there was large heterogeneity in the design of included studies and the reporting of results. Therefore, the number of studies that qualified for inclusion in each specific analysis was often small. Moreover, due to the absence of control groups in all the included studies, a meta-analysis could not be performed. In addition, although only this study provides the systematic review on this subject, a sample size of 111 is limited, which could underestimate the true rate of complications.

## Conclusions

We conclude that IPCs are an acceptable therapeutic option for the management of refractory pleural effusion in patients with HH, and possibly a superior option than TIPS. Although liver transplantation is the gold standard for the management of HH in these patients, availability is a limiting factor. Using IPCs for the management of refractory HH is a safe and efficacious therapy with a good rate of spontaneous pleurodesis.
